# Biosynthesis of silver nanoparticles using *Pseudomonas canadensis*, and its antivirulence effects against *Pseudomonas tolaasii*, mushroom brown blotch agent

**DOI:** 10.1038/s41598-023-30863-x

**Published:** 2023-03-04

**Authors:** Samira Ghasemi, Behrouz Harighi, Morahem Ashengroph

**Affiliations:** 1 Department of Plant Protection, Faculty of Agriculture, University of Kurdistan, Sanandaj, Iran; 2 Department of Biological Sciences, Faculty of Sciences, University of Kurdistan, Sanandaj, Iran

**Keywords:** Biotechnology, Microbiology

## Abstract

This study reports the biosynthesis of silver nanoparticles (AgNPs) using a *Pseudomonas canadensis* Ma1 strain isolated from wild-growing mushrooms. Freshly prepared cells of *P. canadensis* Ma1 incubated at 26–28 °C with a silver nitrate solution changed to a yellowish brown color, indicating the formation of AgNPs, which was confirmed by UV–Vis spectroscopy, scanning electron microscopy (SEM), and X-ray diffraction. SEM analysis showed spherical nanoparticles with a distributed size mainly between 21 and 52 nm, and the XRD pattern revealed the crystalline nature of AgNPs. Also, it provides an evaluation of the antimicrobial activity of the biosynthesized AgNPs against *Pseudomonas tolaasii* Pt18, the causal agent of mushroom brown blotch disease. AgNPs were found to be bioactive at 7.8 μg/ml showing a minimum inhibitory concentration (MIC) effect against *P. tolaasii* Pt18 strain. AgNPs at the MIC level significantly reduced virulence traits of *P. tolaasii* Pt18 such as detoxification of tolaasin, various motility behavior, chemotaxis, and biofilm formation which is important for pathogenicity. Scanning electron microscopy (SEM) revealed that bacterial cells treated with AgNPs showed a significant structural abnormality. Results showed that AgNPs reduced brown blotch symptoms in vivo. This research demonstrates the first helpful use of biosynthesized AgNPs as a bactericidal agent against *P. tolaasii*.

## Introduction

Brown blotch disease is one of the most important diseases in the cultivated mushrooms, *Agaricus bisporus*, in mushroom-growing houses worldwide^1,2^. One of the causal agents of this disease is *Pseudomonas tolaasii*, an endemic bacterium to compost beds and belongs to the order of Gammaproteobacteria and fluorescent group^3^. Brown blotch disease causes considerable losses in the yield of common mushrooms by decreasing the quality and marketability of mushrooms during cultivation or post–harvesting of mushrooms^2^.

*P. tolaasii* produces toxin of lipodepsipeptides class called tolaasin, a virulence factor involved in pathogenesis, which causes symptoms of brown blotch on the mushroom caps from yellowish or light brown to dark brown discoloration^4^. In addition, other compounds such as enzymes and volatile compounds produced by *P. tolaasii* are possibly involved in the infection process and development of blotch symptoms^2,5^. Chemotaxis, motility, and biofilm production are another groups of virulence/survival factors that provide the basis for the development of disease^6^. Due to the limitations in the control of *P. tolaasii* infection in the compost and casing soil^7^, the current method of managing brown blotch disease is mainly related to the prevention of the incidence of infection in farms. However, biological control by using antagonistic bacteria that are native to mushroom substrates or their eco-friendly antibacterial compounds is one of the attractive methods to control *P. tolaasii* brown blotch. It has been reported that bacteria isolated from compost or wild substrates of mushrooms such as *Burkholderia multivorans* A2, *Pseudomonas* spp., *Enterobacter aerogenes* C10, non-pathogenic *P. tolaasii*, *Mycetocola* spp., *Pedobacter* sp. OM-E81, *Sphingobacterium multivorum* OM-A8, *Acinetobacter* sp. OM-H10, *Bacillus pumilus* OM-F6, and *Bdellovibrio bacteriovorus* have the potential to inhibit *P. tolaasii* infection^8–11^. Antivirulence are compounds that target the non-vital activity of bacteria. For this reason, these compounds have advantages such as preventing selective pressure in bacterial population and the emergence of resistant populations^6^.

Recently, nanotechnology science has become an interesting field for plant pathologists to manage plant diseases or promote plant growth^12,13^. Nanoparticles are particles with nanoscale of size ranging from 1 to 100 nm which uses as antimicrobial agents against broad-spectrum microbes including bacterial pathogens^14^. There are different types of metallic nanoparticles e.g. Zn, Mg, Ag, and Fe. Due to the unique physicochemical and biochemical characteristic features of silver nanoparticles (AgNPs), researchers have been focusing on their different applications^15,16^. Owing to their smaller size and large surface area, AgNPs have high electrical, catalytic, optical, and antimicrobial properties^17^. Silver nanoparticles are well-known to inhibit the activity of various microorganisms such as bacteria^18^, viruses^19^ and fungi^20^. Furthermore, metallic nanoparticles such as zinc oxide nanoparticles and Magnesium oxide have been shown high antibacterial activity against *Xanthomonas oryzae* pv. *oryzae* bacterial leaf blight agent^13^. It has been reported that AgNPs could significantly reduce the symptoms of kiwifruit rot caused by *Alternaria alternata, Pestalotiopsis microspora, Diaporthe actinidiae,* and *Botryosphaeria dothidea*^21^.

Traditionally, physical and chemical techniques have been used to synthesize metal nanoparticles. Green synthesis nanoparticles is a novel strategy to synthesize of nanoparticles that it is safe, inexpensive, and eco-friendly compared to other traditional methods^22^. One of the green synthesis techniques is the biological method in which metals ion are reduced by organisms through specific metabolic pathways^23,24^. Microorganisms of different taxonomic categories from fungi and prokaryotes have the potential to produce nanoparticles. These microorganisms produce extracellular substances that consist of polysaccharides, lipids, proteins, and nucleic acids. They contain functional groups that can serve as ligands and binding sites of metals^25^. For example, it has been reported that members of Actinobacteria isolated from various ecological environments e.g. *Thermomonospora* sp., *Rhodococcus* sp., and *Streptomyces* sp. synthesized AgNPs through intra/extracellular^26^. Also, it has been reported that *Fusarium oxysporum*^27^, *Escherichia coli*, *Exiguobacterium aurantiacumm*^28^*, Aspergillus niger*^29^, and *Trichoderma longibrachiatum*^30^*, Bacillus sonorensis*^31^ are able to synthesize silver nanoparticles.

Our previous studies showed that several endofungal bacteria isolated from the wild-growing mushrooms could have antagonistic activity against *P. tolaasii *in vitro and reduce brown blotch symptoms^32,33^. The main purpose of this study was to biologically synthesize AgNPs by these endofungal bacteria through extracellular strategy, optimization and characterization of the synthesized AgNPs, and evaluation of its antimicrobial activity against *P. tolaasii* by investigating bacterial cell growth, effects on virulence traits, observe morphological changes of *P. tolaasii* upon treatment with AgNPs, and further in vivo investigation of its influence on the decrease of brown blotch symptoms.

## Results

### Selection of bacterial isolate capable of synthesizing AgNPs

**Figure 1:**
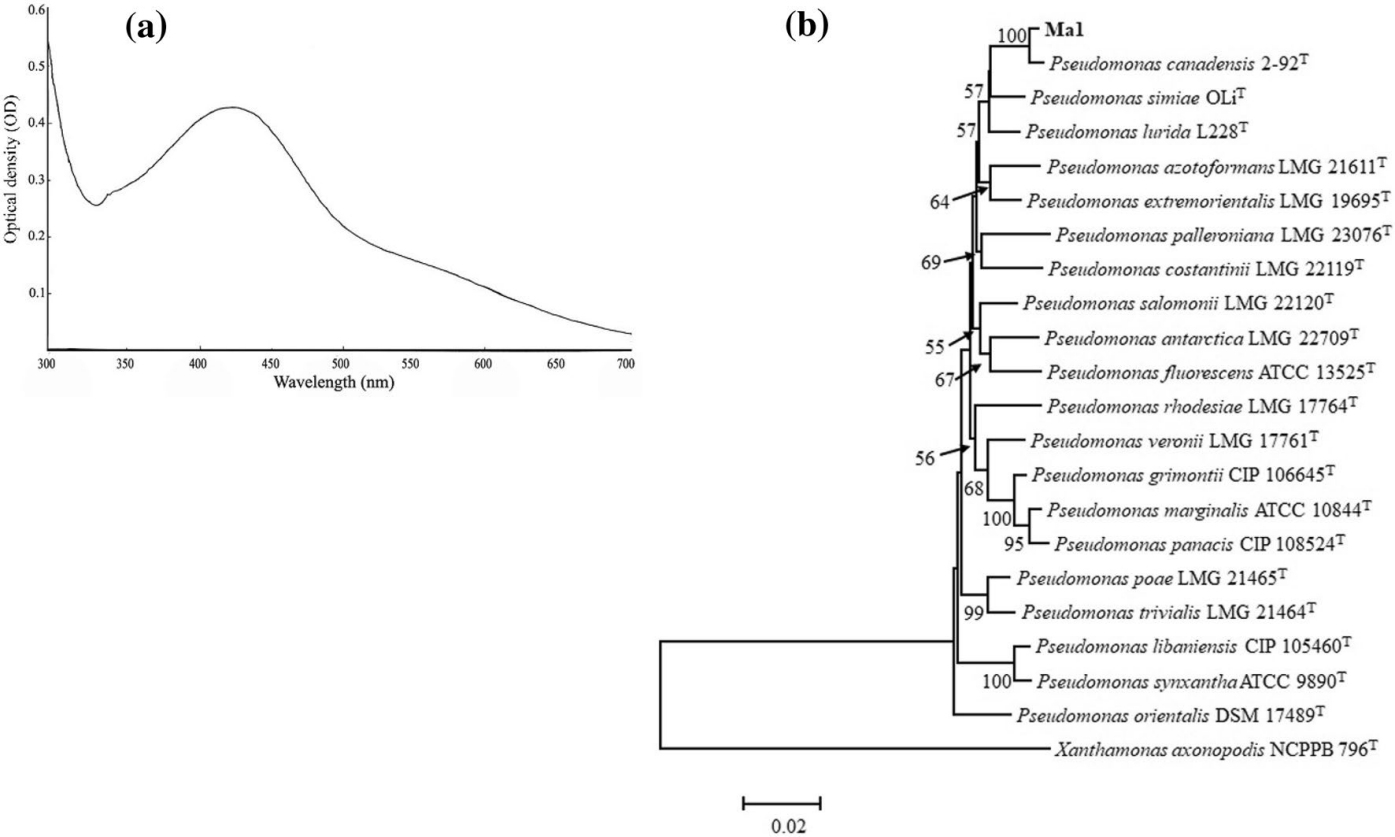
(**a**) The UV–Visible absorption spectrum of the AgNPs solution synthesized with *Pseudomonas canadensis* Ma1. AgNPs display a clear absorbance peak at 400–450 nm, and (**b**) Phylogenetic tree of partial *16S rRNA* and *rpoD* gene sequences showing the position of Ma1 strain (shown in bold) in addition to taxonomically similar selected reference strains. The analysis was conducted by the Maximum Likelihood method with Tamura-Nei calculation model in MEGA version 6.0. The scale bar represents the number of substitutions per site. Numbers at branching points indicate bootstrap value derived from 1000 replicates.

To select an isolate capable of synthesizing silver nanoparticles, bacterial cells were treated with AgNO_3_ (1 mM). The synthesis of silver nanoparticles was monitored based on the change of color from light yellow to dark brown and further confirmed by UV–Vis spectroscopy. In a comparison to other isolates, the treated suspension of isolate Ma1 showed a color change to brown and an absorption peak of AgNPs at the wavelength range from 400 to 450 nm was the characteristic of silver nanoparticles (Fig. 1a).

### Molecular identification of bacterial isolate Ma1

Based on the phenotypic analysis, the Ma1 isolate was Gram-negative, facultatively anaerobic, oxidase negative, catalase positive, and producing fluorescent pigment. Nucleotide sequences obtained were submitted to the NCBI nucleotide sequence database under accession numbers OP748753 and OP886707 for the *16S rRNA* and *rpoD* genes, respectively. Results of the partial nucleotide sequencing of the *16S rRNA* and *rpoD* genes revealed that bacterial isolate clustered into the *Pseudomonas* genus. Blast search results revealed that strain Ma1 had 99% similarity to *Pseudomonas canadensis*. The phylogenetic tree was constructed which shows the position of Ma1 strain amongst the type strains of the genera *Pseudomonas* species (Fig. 1b).

### Optimization process

**Figure 2:**
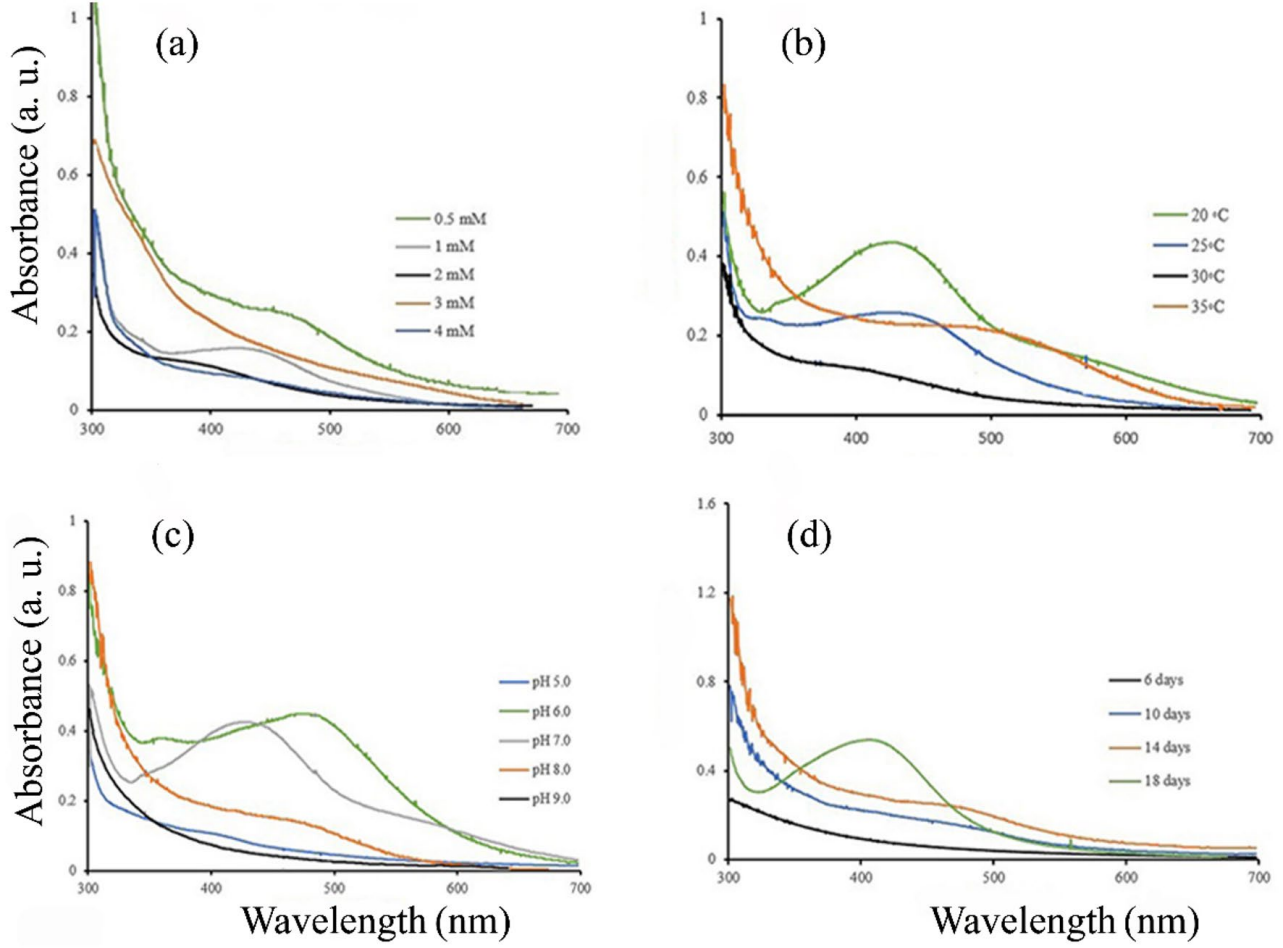
Optimization process of the nanoparticle by measuring UV–visible absorption spectra of biosynthesized AgNPs at different (**a**) AgNO_3_ concentrations, (**b**) temperatures, (**c**) pH, and (**d**) incubation times.

Synthesis of silver nanoparticles produced by *P. canadensis* was optimized with four factors, concentration of AgNO_3_, temperature, pH, and incubation time by using one variable at a time approach method. The results showed that the optimum concentration of AgNO_3_ to produce AgNPs was predicted as 1 mM by UV–visible spectra at the maximum absorbance peak of 425 nm. ANOVA results show a significant difference among different concentrations (F = 1314.33, p < 0.0001) (Table 1). At AgNO_3_ concentrations of 3 and 4 mM, synthesis of AgNPs was not observed (Fig. 2a).

**Table 1:** Analysis of variance (ANOVA) of optimization process of AgNPs biosynthesis.

Source of variation	Source of variation			
	Concentration	Temperature	pH	Incubation time
Treatments	0.07	0.003	0.06	0.04
Error	0.00006	0.00003	0.00002	0.003
F-value	F_2,6_ = 1314.33**	F_1,4_ = 103.85**	F_2,6_ = 2669.84**	F_3,8_ = 11.54**
CV%	2.23	1.15	1.53	19.71

**Significant at 5% probability level.

The effect of varying temperatures on AgNPs production by *P. canadensis* Ma1 was carried out at different temperatures from 20 °C to 35 °C with a difference of 5 °C. In a comparison to other temperatures (F = 103.88, p < 0001), the maximum synthesis of AgNPs was attended at 20 °C (at the optimized concentration of 1 mM), and detected by UV–visible absorption spectra the maximum peak was at 425 nm (Fig. 2b). Silver nanoparticles were not synthesized at 30 °C and 35 °C.

In a concentration of 1 mM and temperature of 20 °C, the synthesis of AgNPs due to the effect of varying pH from 5 to 9 was depicted by UV–visible absorption spectra. The maximum peak at pH 7 was about 425 nm and higher than the others (F = 103.85, p < 0.0001) (Fig. 2c). Under the alkaline conditions, the synthesis of AgNPs by *P. canadensis* Ma1 was not observed. The results of optimizing of incubation time showed density of AgNPs gradually increased from 10 to 18 days (Fig. 2d). There was significant difference among incubation times (F = 11.54, P < 0028).

### Characterization of biosynthesized AgNPs

**Figure 3:**
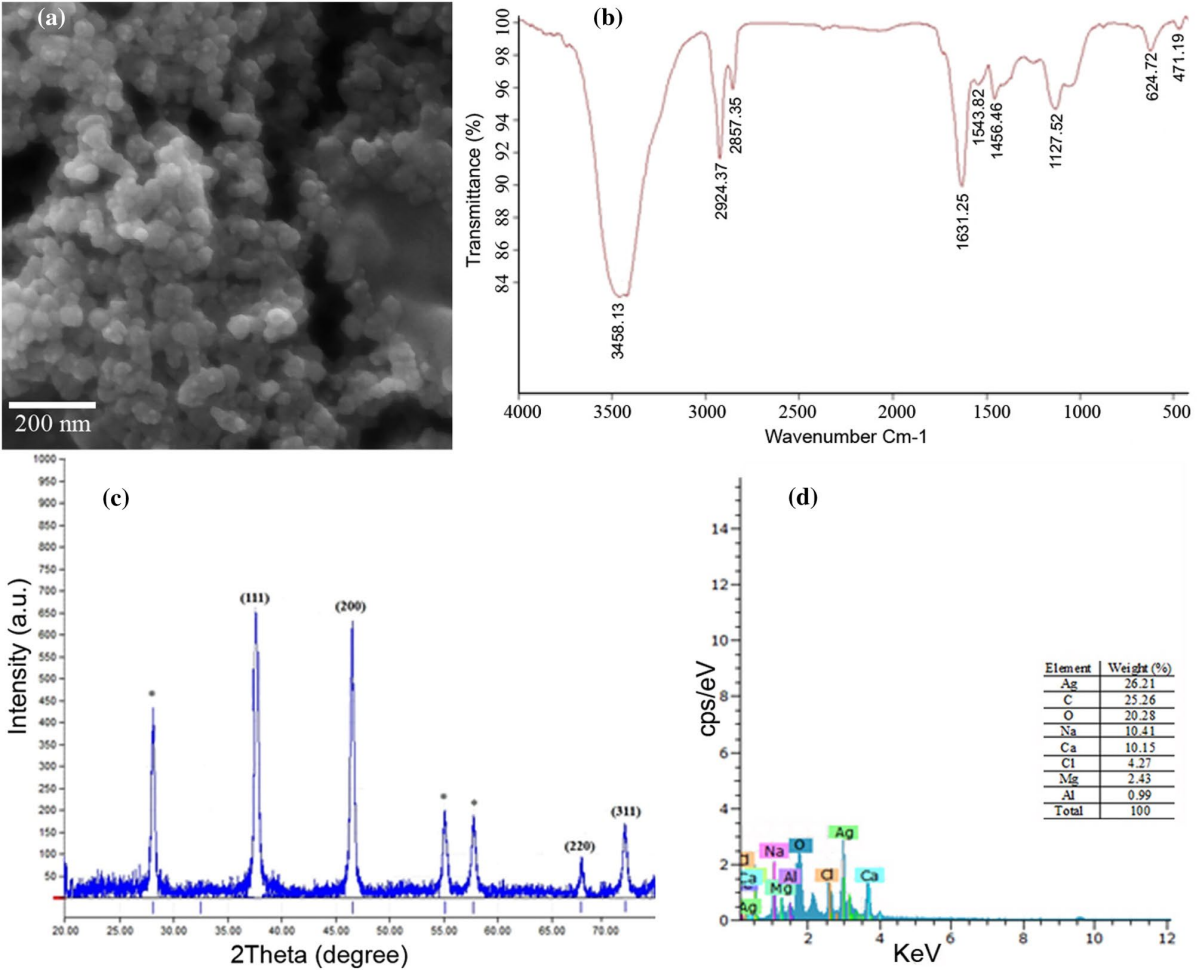
Characterization of AgNPs synthesized with *Pseudomonas canadensis* Ma1. (**a**) Scanning electron micrograph showing AgNPs in spherical forms about 21‒52 nm in diameter, (**b**) Fourier transform infrared (FTIR) spectrum showing functional groups responsible for the synthesis and stabilization of AgNPs, (**c**) X-ray diffraction (XRD) spectrum showing the nanoscale size and crystalline nature of the AgNPs, the unassigned peaks is labeled with (*), and (**d**) Energy dispersive spectrum (EDS) pattern showing the predominance of Ag element in the AgNPs product.

The results of SEM revealed that the biosynthesized AgNPs were spherical in shape. The size of particles ranged from 21 to 52 nm with an average of 32 nm (Fig. 3a). To characterize functional groups in the biosynthesized AgNPs, FTIR analysis was done. The FRIR spectrum shows absorption peaks at 3458.13, 2924.37, 2857.35, 1631.25, 1543.82, 1456.46, 1127.52, 624.72, and 471.19 cm^−1^ (Fig. 3b). The major peak at 3458.13 may be due to –OH stretching from polysaccharides, the peaks at 2924.37 and 2857.35 are related to the C–H stretching of alkanes. The band at 1631 cm^−1^ in the spectra corresponds to the C = O stretching of the peptide bond. The peaks at 1543.82 and 1456.46 are attributed to N–H and C–O of aromatic amine, respectively. The absorption in the 1127.52 cm^−1^ regions was corresponding to the O–H group.

The crystalline structure of biosynthesized AgNPs was confirmed by XRD spectrum analysis with the standard silver (JCPDS file no. 04–0783). XRD analysis showed the diffraction peaks of the Ag nanocrystallites at 2θ values of 37.52°, 46.52°, 67.68°, and 74.4° corresponding to 111, 200, 220, 311 planes. The unassigned peaks with the (*) symbol were recorded at 28.2°, 55.06°, and 57.73° that are related to the organic phase or silver oxide (Fig. 3c).

The Elemental analysis of the biosynthesized AgNPs was performed by EDS over the SEM. The EDS analysis showed that the AgNPs display a typical absorption peak of Ag at 3 keV associated with the reduction of Ag + to Ag^0^. The other signals, Ca and Cl atoms, are most likely caused by X-ray emission from the remaining media. Signals of C and O atoms may be related to capping protein (Fig. 3d).

### Antibacterial assay, minimum inhibitory concentration (MIC) and minimum bactericidal concentration (MBC)

**Figure 4:**
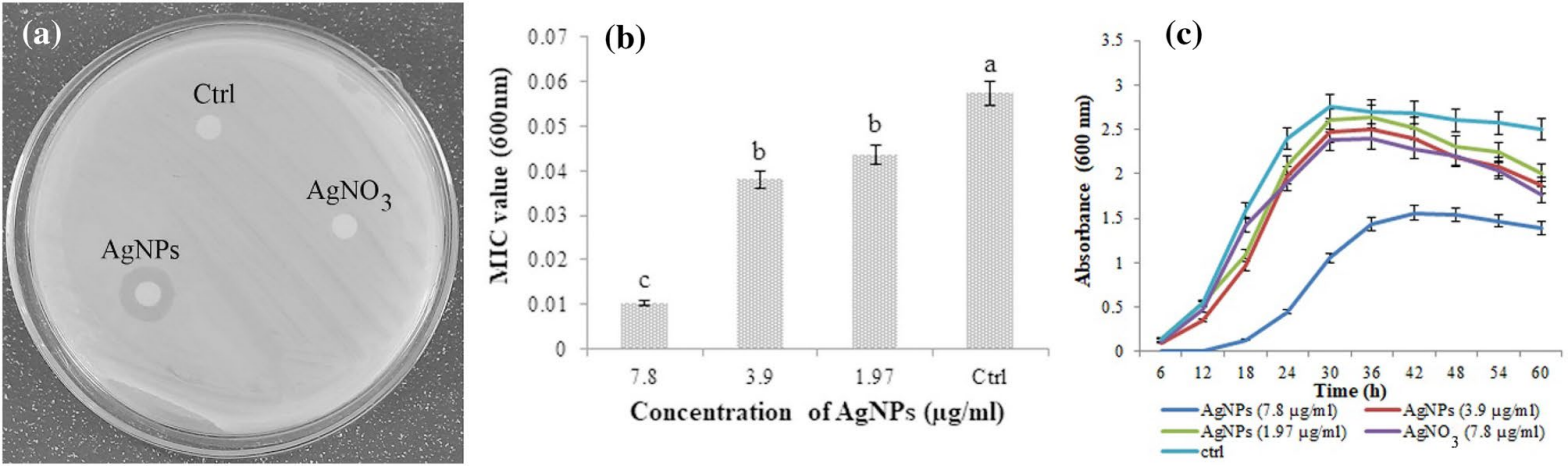
Antibacterial activity of AgNPs against *P. tolaasii* Pt18, (**a**) measuring inhibition zone sizes around paper disc impregnated with biosynthesized AgNPs, AgNO_3_, and the cell-free supernatant filtrate of *P. canadensis* Ma1 (Ctrl), (**b**) Minimum inhibitory concentration (MIC) values of AgNPs. Data are presented as mean values with standard errors. Values represented with different letters indicate significant differences between treatments (P = 0.05). At higher concentrations of 7.8 µg/ml, absorption in 600 nm was not recorded. (**c**) Growth kinetics curve of *P. tolaasii* Pt18 in the presence of 7.8 µg/ml concentration of AgNPs as well as pure silver nanoparticles, and control (Ctrl).

The antibacterial activity of biosynthesized AgNPs was investigated against *P. tolaasii* Pt18 using the paper disk method. As shown in Fig. 4a, a paper disk impregnated with cell-free supernatant containing AgNPs generated a clearing inhibition zone with a diameter of 9 mm. The cell-free supernatant filtrate of *P. canadensis* Ma1 (Ctrl) and filter paper disk impregnated with silver nitrate solution (1 mM) did not show any inhibitory activity against *P. tolaasii* Pt18 growth. To determine the MIC of AgNPs, as the lowest concentration of the antibacterial agent to inhibit the growth of bacteria, various concentrations of AgNPs (1000 ~ 1.97 µm/l) were used against *P. tolaasii* Pt18. The MIC value of AgNPs was 7.8 μg/ml against *P. tolaasii* Pt18. Lower concentrations did not show a significant difference in to control (Fig. 4b) (F = 42.92, p < 0001).

The biosynthesized AgNPs showed an MBC value of 15.62 µg/ml. In lower concentrations of AgNPs, growth of *P. tolaasii* Pt18 was observed on Agar plates. The growth inhibitory effect of 7.8, 3.9, and 1.97 µg/ml concentrations of AgNPs against *P. tolaasii* Pt18 is shown in Fig. 4c. By measuring absorption in 600 nm, no growth of the bacterial cell was monitored at 7.8 µg/ml during 6 and 12 h compared to the growth of *P. tolaasii* Pt18 in AgNO_3_ and NB medium as controls. After 18 h, the growth rate of *P. tolaasii* Pt18 medium supplemented with AgNPs (7.8 µg/ml concentration) was significantly reduced than other concentrations and controls.

### Tolaasin detoxification assay

**Figure 5:**
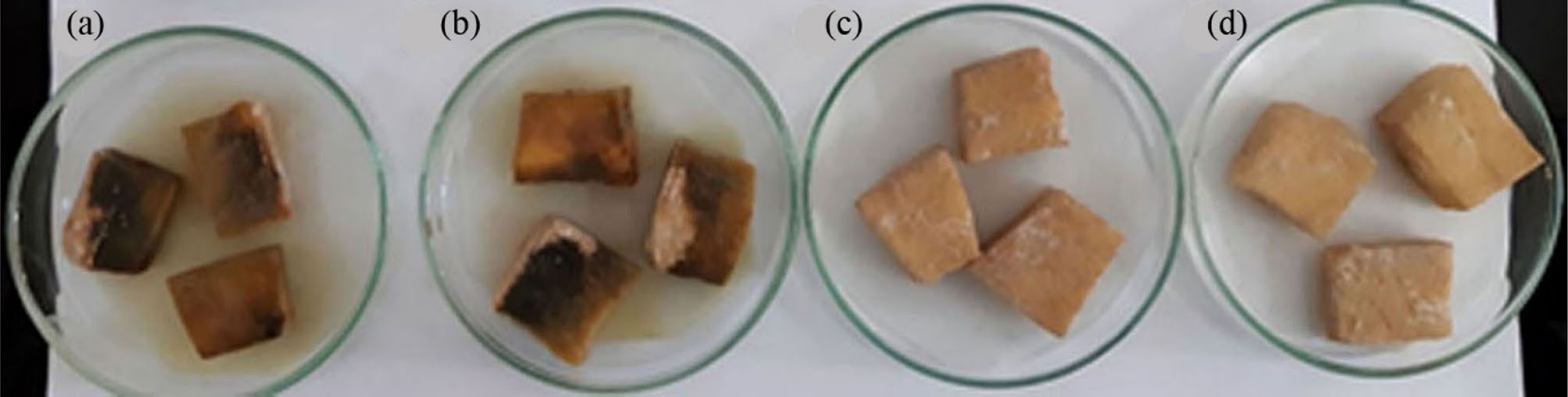
Effect of AgNPs on detoxification of tolaasin produced by *Pseudomonas tolaasii* Pt18 in potato tuber slices inoculated with (**a**) PSB-Tol, (**b**) PSB-Tol + AgNO_3_, (**c**) PSB-Tol + AgNPs, and (**d**) AgNPs.

Results of the assay showed that when PSB-Tol supplemented with AgNPs were inoculated, blackening did not develop after 48 h, whereas potato slices inoculated with PSB-Tol alone, and PSB-Tol supplemented with AgNO_3_ developed complete blackening during the same period of time (Fig. 5).

### Motility assay

**Figure 6:**
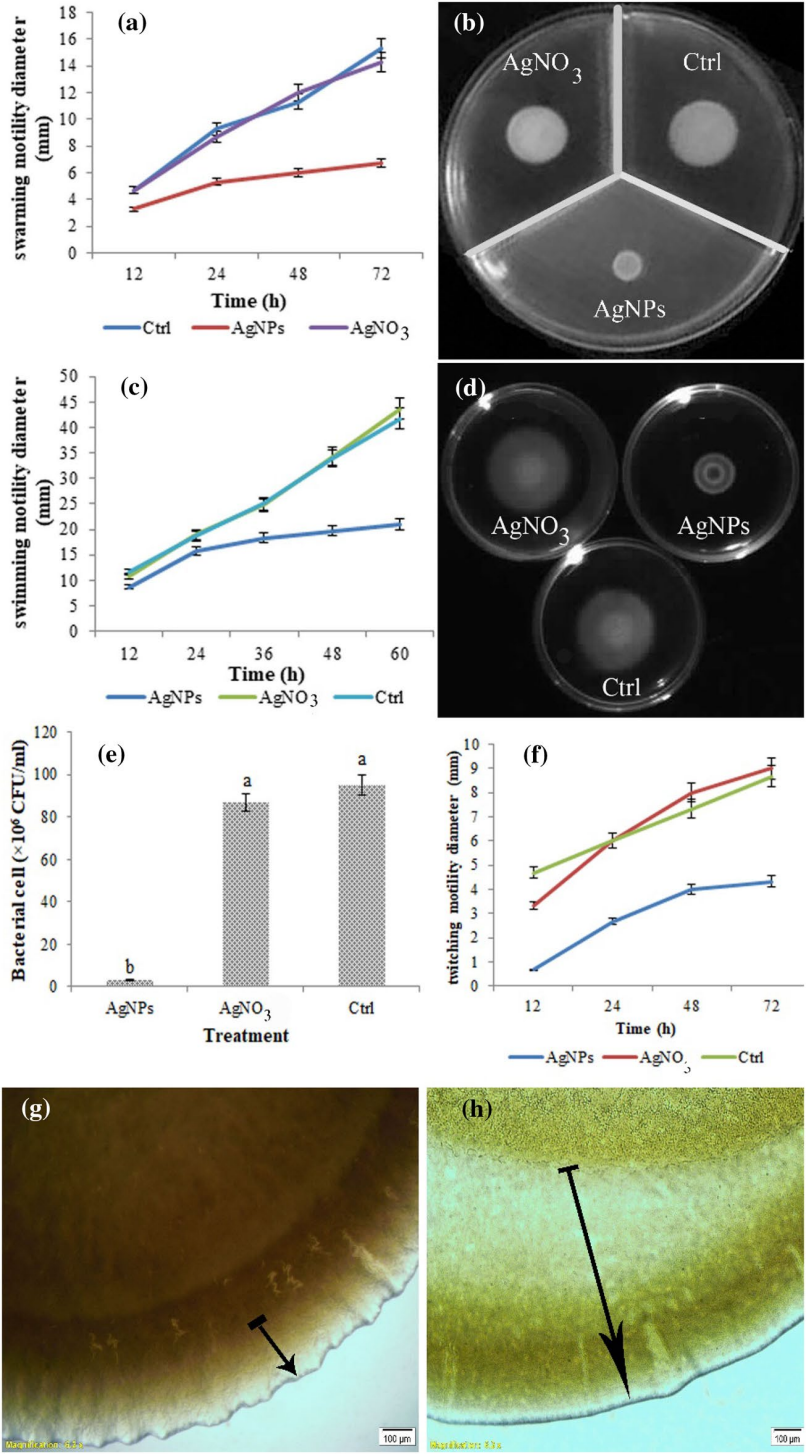
Effects of AgNPs biosynthesized by *Pseudomonas canadensis* Ma1 on various motilities of *P. tolaasii* Pt18 compared to the untreated control (Ctrl). (**a**) the diameter of the motility zone, and (**b**) a representative plate of the swarming motility assay, (**c**) the diameter of the motility zone, and (**d**) a representative plate of the swimming motility assay, (**e**) the chemotaxis behavior toward mushroom extract, (**f**) the diameter of twitching motility zone, and representative microscopic examination of the peripheral edge of twitching motility, (**g**) bacterial cells treated with AgNPs, (**h**) untreated control (Ctrl). Arrowheads indicate a twitching zone. Three replicates were used for each treatment. Error bars indicate the SE of the three replicates. Different letters indicate significant differences (p = 0.05).

The effects of 7.8 µg/ml of AgNPs on the three types of *P. tolaasii* Pt18 motility including swarming, swimming, and twitching was measured. ANOVA results showed that AgNPs significantly inhibited the swarming, swimming, and twitching motility of *P. tolaasii* Pt18 compared to the control (Table 2). As shown in Fig. 6a and b, the swarming motility of bacterial cells in the presence of AgNPs was the mean of 6.7 mm compared to the control with the mean of 15.3 mm (F = 202.33 p < 0.0001).

**Table 2:** Analysis of variance (ANOVA) of chemotaxis, swimming-, swarming- motility, biofilm production and discoloration level (ΔE) of mushroom cap inoculated by *Pseudomonas tolaasii* Pt18 under the effect of biosynthesized AgNPs.

Source of variation	Source of variation					
	MIC	Swimming	Swarming	Chemotaxis	Biofilm	ΔE
Treatments	0.0011	472.44	67.44	7.82	0.03	1476.59
Error	0.00002	1.22	0.33	1.18	0.01	7.18
F-value	F_3,8_ = 42.92**	F_2,6_ = 386.55**	F_2,6_ = 202.33**	F_2,6_ = 66.37**	F_2,6_ = 71.66**	F_5,12_ = 203.92**
CV%	13.98	3.11	4.76	17.58	10.42	12.19

**Significant at 5% probability level.

Similarly, the swimming motility zone of bacterial cells in the absence of AgNPs was the mean of 41.7 mm, which reduced to the mean of 21.00 mm after AgNPs were added to the medium (F = 386.55, P < 0.0001) (Fig. 6c,d).

In the chemotaxis assay, the result of ANOVA (Table 2) showed that significant differences exist between treatments (F = 66.37; p < 0.0001). *P. tolaasii* Pt18 cells showed significantly higher motility toward the hole containing mushroom extract in the control (Fig. 6e). In the presence of AgNPs the chemotaxis activity of *P. tolaasii* Pt18 reduced to about 96.2% compared to the non-treated control.

Results of twitching motility revealed that in the presence of AgNPs colony diameter was reduced to the mean of 4.33 mm compared to the control with the mean of 9 mm (Fig. 6f). Microscopic examination of the twitching motility showed that the peripheral colony edge of *P. tolaasii* Pt18 in the control was considerably wider than those treated with AgNPs (Fig. 6g,h).

### Morphological changes

**Figure 7:**
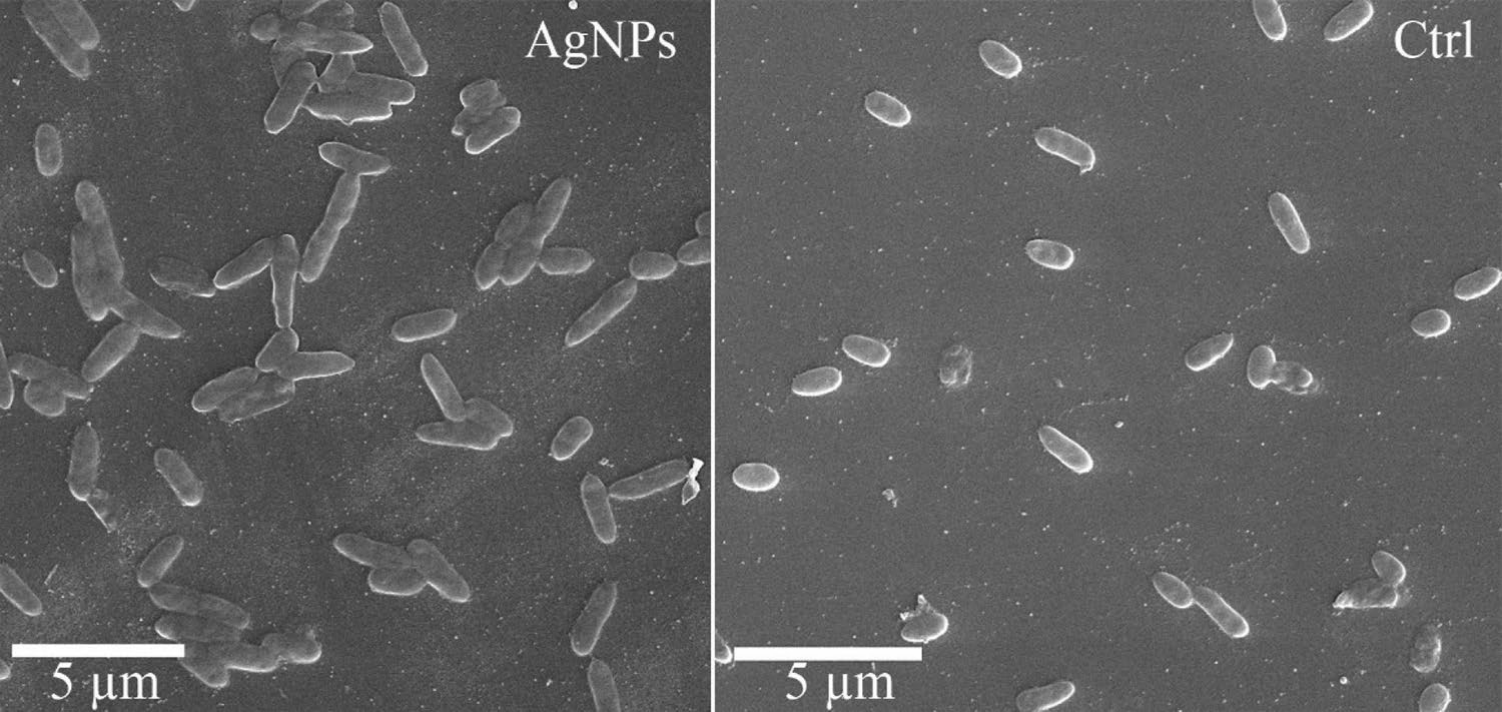
Scanning electron micrographs of *Pseudomonas tolaasii* Pt18 cells treated with AgNPs biosynthesized by *Pseudomonas canadensis* Ma1 strain. (Ctrl) non-treated control cells.

The morphological change potential of AgNPs against *P. tolaasii* Pt18 was viewed with SEM. The surface of bacterial cells grown in the absence of AgNPs was smooth and showed normal cell structure, while alteration in length and some destruction cells were observed following treatment with AgNPs (Fig. 7).

### Biofilm formation assay

**Figure 8:**
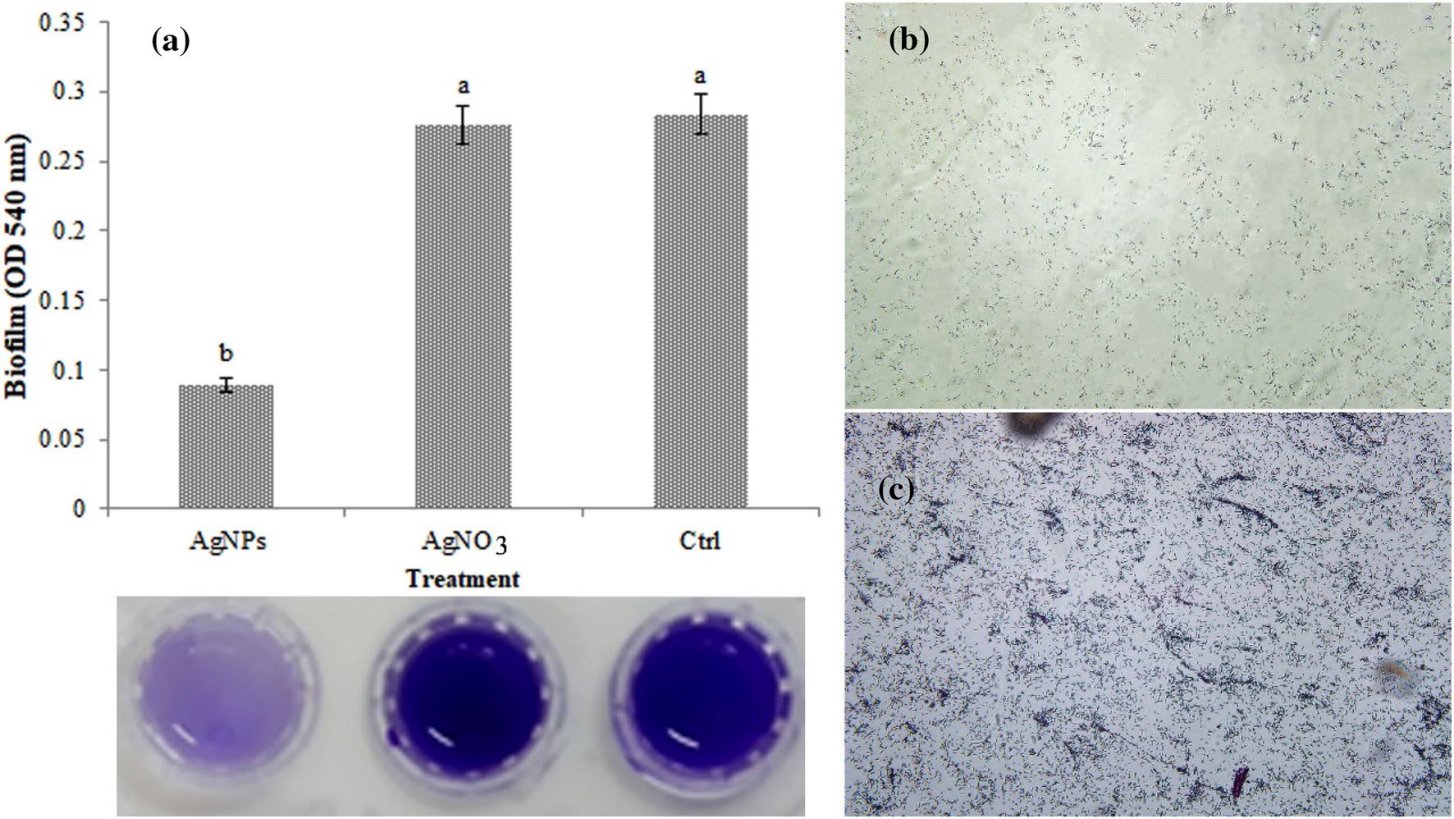
Effect of AgNPs biosynthesized by *Pseudomonas canadensis* Ma1 on (**a**) biofilm formation of *Pseudomonas tolaasii* Pt18, and representative bacterial cells staining (**b**) treated with AgNPs, and (**c**) untreated control. The graph represents the mean of three replicates. Error bars indicate the SE of the three replicates. Different letters indicate significant differences (p = 0.05).

The biofilm formation was performed using polypropylene tubes. The results showed that AgNPs produced by strain *P. canadensis* Ma1 had a significant inhibition effect on the biofilm formation by *P. tolaasii* Pt18 (Table 2) (F = 71.66, p < 0001). As shown in Fig. 8a, compared to untreated control, in the presence of AgNPs more than 68% reduction in biofilm formation by *P. tolaasii* Pt18 was observed. These results were further verified with optical microscopy (Fig. 8b,c).

### Effect of biosynthesized AgNPs on brown blotch disease development

**Figure 9:**
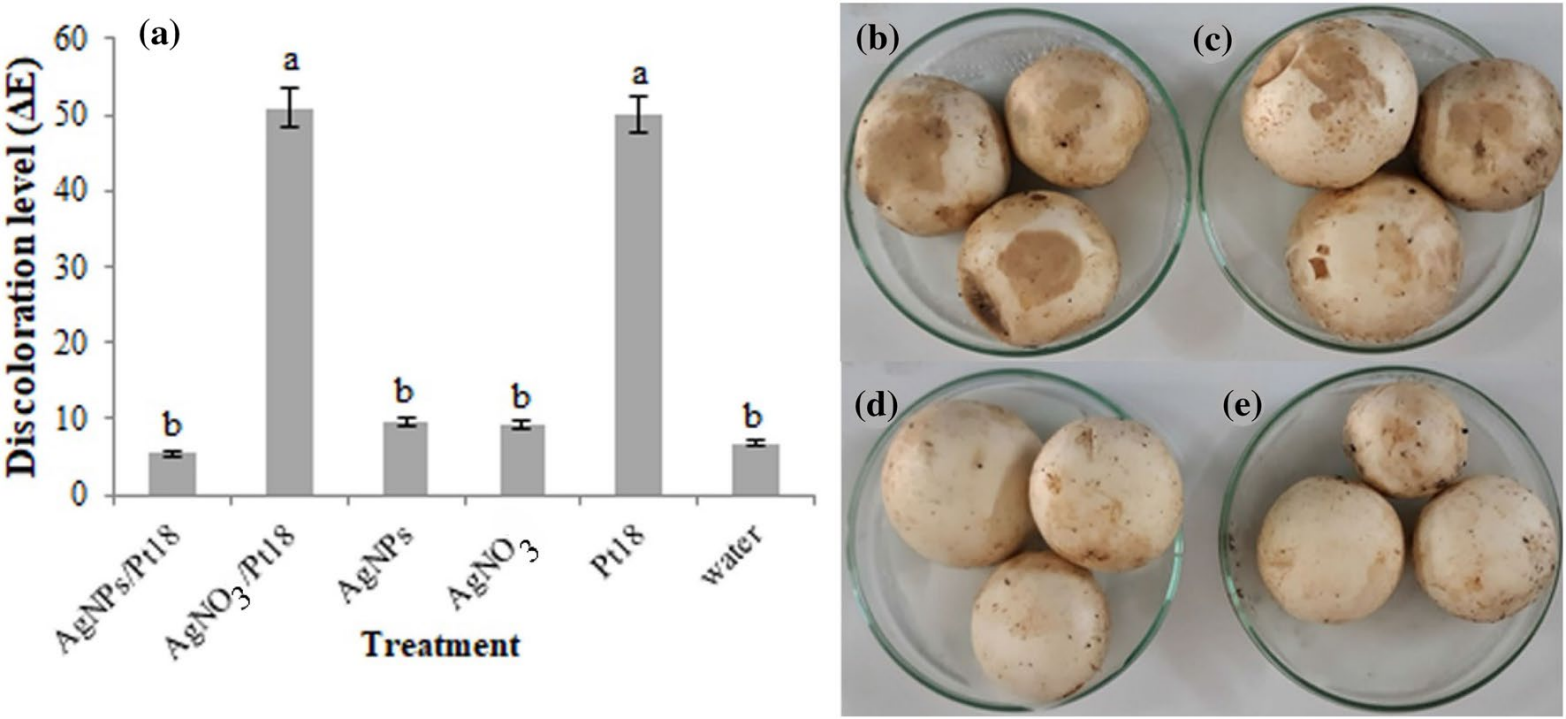
Effect of AgNPs biosynthesized by *Pseudomonas canadensis* Ma1 on (**a**) discoloration level (ΔE) of *Agaricus bisporus* caps inoculated with *Pseudomonas tolaasii* Pt18 (Pt) compared with controls. Representative mushrooms caps inoculated with (**b**) *P. tolaasii* Pt18 alone, (**c**) AgNO_3_, (**d**) AgNPs, and (**e**) sterile distilled water are shown. The graph represents the mean of three replicates. Error bars indicate the SE of the three replicates. Different letters indicate significant differences between treatments (p = 0.05).

The effect of AgNPs on reducing brown blotch symptoms was calculated by measuring the discoloration of mushroom caps inoculated with *P. tolaasii* Pt18 in the presence/absence of AgNPs. Based on the results of ANOVA, significant differences exist among treatments in terms of discoloration level (ΔE) (F = 203.00, p < 0001). As shown in Fig. 9a, *P. tolaasii*-inoculated mushrooms showed the highest color changes in all parameters measured compared to pretreated mushrooms with AgNPs or control. The results of discoloration level (ΔE) showed that AgNPs were able to significantly decrease the browning of mushroom caps to about 95% compared to caps inoculated with *P. tolaasii* Pt18 alone or treated with AgNO_3_ (Fig. 9b–e).

## Discussion

In the present study, we identified bacterial strain previously isolated from wild-growing mushrooms and subsequently evaluated themfor the biosynthesis of AgNPs using different concentrations of AgNO_3_. Based on molecular analysis this strain was identified as *Pseudomonas canadensis* Ma1. This species was previously reported with in vitro antagonistic activity against fungal pathogens^34^. There has been no report of the use of bacteria closely related to *P. canadensis* for the synthesis of AgNPs. Although, other taxonomically related strains like *P. hibiscicola*, *P. aeruginosa*, and *P. stutzeri* have been reported for the synthesis of silver nanoparticles^35–37^.

The biosynthesized AgNPs showed a peak at 425 nm in UV/Visible spectral analysis. This result is in agreement with previous studies that confirmed the formation of AgNPs by other bacteria with the presence of the peak at 400–450 nm^35^. Analysis through an XRD spectrometer confirmed the presence of an elemental silver signal of the AgNPs. XRD spectrum compared with the standard showed the diffraction peaks of the Ag nanocrystals as evident from the peaks correspondence to 111, 200, 220, 311 planes, which have been indexed for silver^38^. The morphological characteristic of the biosynthesized nanoparticles was identified using SEM analysis. The particles were spherical in shape with the size ranging from 21 to 52 nm. These results are consistent with previous reports. However, slight differences in nanoparticle size and chemical composition of AgNPs exist between this study and other previous reports^39–41^.

The presence of functional groups in *P. canadensis* Ma1 suspension responsible for the reduction of Ag + and stabilization of the AgNPs was identified by FTIR spectroscopy. The FTIR spectrum shows absorption peaks corresponding to several functional groups that confirm the presence of proteins and polysaccharides in bacterial suspension, suggesting that have a role in reducing Ag + for the synthesis of AgNPs and binding to nanoparticles for its stabilization as reported earlier^41^. The predominance of Ag element in the AgNPs product was confirmed by energy dispersive spectroscopy. The AgNPs generally display a typical absorption peak of Ag element at about 3 keV which is in agreement with previous studies^42^.

AgNPs have shown excellent antibacterial activity against a wide range of bacteria^43^. In the present study, the biosynthesized AgNPs have been evaluated for their antibacterial activity against mushroom brown blotch pathogen. The results of in vitro experiment showed that the biosynthesized AgNPs at different concentrations had antibacterial activity against *P. tolaasii* Pt18 with MIC of about 7.8 µg/ml. The inhibitory effect increased with the increase of AgNPs concentration. This result is in agreement with previous studies indicating the bactericidal effect of AgNPs against several bacterial plant pathogens, like *Dickeya dadantii*, *Pseudomonas syringae* pv. *tomato*, *Xanthomonas campestris* pv. *campestris*, *Clavibacter michiganensis* subspecies *michiganensis*, *Ralstonia solanacearum*, and *Xanthomonas oryzae* pv. *oryzae*^44–47^. The antibacterial effects of AgNPs might be through the NPs or released Ag^+^. Compared to our study, Yang et al.^48^ demonstrated that ZnO-NPs had a dose-dependent inhibition effect on the growth of *P. tolaasii* Y-11 via NPs themselves and the released Zn^2+^. In agreement with the result of in vitro inhibition effect, the biosynthesized AgNPs significantly reduced mushroom brown blotch symptoms in vivo as compared to the control.

Our SEM observation indicated structural changes in bacterial cells treated with AgNPs. This result showed that the inhibitory effect of AgNPs on mushroom brown blotch agent might has been at least in part due to damage to the bacterial cells. The destruction effect on bacterial cells by AgNPs might be attributed to an accumulation of nanoparticles in the bacterial membrane or in the cytoplasm, which eventually leads to cell death. In agreement with the present finding, the destructive effect of AgNPs on the structure of several bacterial cells is well documented^44^.

Chemotaxis and motility toward *Agaricus bisporus* exudate are important for pathogenicity in *P. tolaasii*^49^. Motility is also involved in host cell adhesion and biofilm formation, two essential steps to initiate brown blotch disease development by *P. tolaasii*^2^. Therefore, it is essential to understand whether AgNPs affect the motility behavior of *P. tolaasii* cells. In the present study, *P. tolaasii* Pt18 showed a significant reduction in swarming-, swimming-, twitching-motility, and chemotaxis toward mushroom extract after treatment with AgNPs, which can be attributed to the bactericidal activity of the nanoparticles. In other studies contrasting results were reported when testing the effects of AgNPs on different bacteria. At concentrations lower than MIC reduction in swarming motility was obtained by Hussain et al.^50^. Another study showed that subinhibitory concentrations of AgNPs significantly increased the swarming, swimming, and twitching motility of *P. aeruginosa*^51^. These differences may be related to AgNPs concentrations or nanoparticle size.

Bacteria communicate with each other by intimate contact, forming well-organized three-dimensional structures, referred to as biofilms. Biofilm formation is an important component of bacterial ecology and fundamental processes during host colonization and infection^52^. The result of the anti-biofilm assay demonstrated that AgNPs significantly inhibited the formation of biofilm by *P. tolaasii* Pt18 compared to the control or AgNO_3_. Previous studies indicated that AgNPs are able to penetrate into the bacterial biofilm and destroy its structure^53^. The in vivo reduction of mushroom brown blotch symptom development was consistent with the in vitro inhibition of bacterial growth, various motility behaviors, and biofilm formation. AgNPs likely inhibit virulence traits of bacterial pathogens via adhering to the bacterial cell surface, releasing toxic Ag +, and damaging cell membranes^54^. Based on the results presented in this study, AgNPs adhering to bacterial cell surfaces may inhibit *P. tolaasii* Pt18 movement and adhesion to mushroom surfaces, following the release of Ag + may destroy bacterial cells.

In conclusion, the aim of the present study was to identify bacterial strains capable of biosynthesis of AgNPs. The biosynthesized AgNPs were further confirmed and characterized by analysis of UV–visible spectroscopy, FTIR, XRD, SEM, and EDS. Results presented in this study confirmed the antibacterial action of biosynthesized AgNPs against *P. tolaasii*, which causes destructive bacterial brown blotch disease. Our results demonstrate that the antibacterial activity of the biosynthesized AgNPs may be at least partially attributed to their inhibition of bacterial growth and its reduction effects on virulence traits such as detoxification of tolassiin, biofilm formation, chemotaxis, and various motility behaviors. SEM imaging demonstrated that morphological abnormality is an important toxicity mechanism. For in vivo application, the control efficacy of AgNPs reached 90%. Taken together, these results demonstrated that AgNPs have great potential as biocides for managing brown blotch disease. AgNPs are used as antimicrobial agents in agriculture and biotechnology. They have great bactericidal potential against a wide range of pathogens. Therefore, further research is needed on the action of AgNPs in the virulence mechanism of *P. tolaasii* for a better understanding of their effects. These results are an initial contribution to broadening our understanding of the effects of AgNPs on *P. tolaasii* behavior. Moreover, it is necessary to explore different concentrations of AgNPs used with the aim of reducing environmental impacts and human health.

## Methods

### Bacterial strains and growth conditions

The endofungal bacteria previously isolated from wild-growing mushrooms, as well as *Pseudomonas tolaasii* Pt18 (GenBank Acc. No. KY203807), which exhibited brown blotch disease in *Agaricus bisporus* obtained from the department of plant protection, University of Kurdistan were used in this study^55^. Accordingly, endofungal bacteria and bacterial pathogen were grown on nutrient agar (NA) medium and then stored at 4–6 °C as a working stock or grown in Luria–Bertani (LB) medium for 24 h at 26–28 °C with shaking. Finally, sterile glycerol was added to the final concentration of 15% and then stored at − 20 °C.

### Plant and mushroom materials

Potato tuber and Mushrooms were kindly provided by the department of Plant Production and Genetics, and department of Horticultural science, University of Kurdistan, Iran. For the collection of plants and mushrooms, all relevant permissions have been obtained where applicable. The experimental research on potato tubers and mushrooms conducted in this study complies with relevant institutional, local, and national regulations.

### Selection of bacterial isolate capable of synthesizing AgNPs

The biosynthesis of silver nanoparticles was carried out based on the procedure previously described^56^. Endofungal bacterial strains were cultured in 100 ml of LB medium at 26–28 °C and at 150 rpm. The bacterial cells were obtained by centrifuging (7000 rpm, at 4 °C for 5 min), and washed three times with Milli-Q water. Then, the cell pellets (concentration of about 20 g/L) were suspended in the sterilized Milli-Q^®^ water and AgNO_3_ was added to a final concentration of 1.0 mM, kept in the dark, and shaken at 150 rpm and 26–28 °C. After incubation time, the biosynthesis of AgNPs was monitored by observing the color change of the solution (from yellow to brown) and analyzed by UV–Visible spectrometer (SPECORD 210, Analytik Jena, Germany) at 300–700 nm. The non-treated suspension was used as a control.

### Molecular identification of bacterial isolate Ma1

The preliminary phenotypic characterization of endofungal bacterial strain Ma1 capable of biosynthesized AgNPs was performed based on the methods previously described^57^. This strain was further identified by partial nucleotide sequencing of the *16 s rRNA* gene using PCR with two universal primers, fD2 (5′-AGA GTT TGA TCA TGG CTC AG-3', position 8–27) and rP1 [5′-ACG GTT ACC TTG TTA CGA CTT-3', position 1512–1492 (*Escherichia coli*)]^58^, and *rpoD* gene using primers PsEG30F (5'- ATYGAAATCGCCAARCG-3') /PsEG790R (5'-CGGTTGATKTCCTTGA-3') based on the method previously described^59^. The amplification conditions included a denaturation period at 94 °C for 5 min followed by 30 cycles of amplification (denaturation was performed at 94 °C for 1 min, primer annealing was performed at 55 °C for 1 min, and primer extension was performed at 72 °C for 1.5 min). A final elongation step was carried out at 72 °C for 10 min. The PCR products were sequenced using an ABI3730XL DNA sequencer (Applied Biosystems). Both sequences obtained were aligned and manually adjusted where necessary by using BioEdit Sequence Alignment Editor 7.0.9.0 software^60^. The *16S rRNA* and *rpoD* gene sequences were further subjected to BLAST analysis with other sequences deposited in the NCBI database using BlastN program. The Maximum Likelihood phylogenetic analysis was performed using MEGA version 6.0^61^ and a phylogenetic tree was constructed (bootstrap analysis with 1000 replicates was conducted).

### Optimization process

An optimization process was carried out with the purpose of investigating the optimal values of effective factors on the extracellular fabrication of AgNPs, including the silver nitrate concentrations (0.5, 1.0, 2.0, 3.0, and 4.0 mM), a reaction temperature (20, 25, 30, and 35 °C), pH of reaction (5, 6, 7, 8, and 9), the incubation time (6, 10, 14 and 18 days) using the one-factor-at-a-time method, under resting cell strategy. All tests were repeated three times. For the recovery of AgNPs, the final dark brown solution was centrifuged (at 4 °C at 14,000 rpm for 20 min). The pellet containing AgNPs was washed three times with Milli-Q water and dehydrated by freeze-drying process (Christ Alpha 1- 2Dplus, Germany) at − 40 °C for 3 h.

### Characterization of biosynthesized AgNPs

Field emission scanning electron microscopy (FESEM) equipped with an Energy Dispersive Spectroscopy (EDS) (TESCAN Mira 3-LMu, Czech Republic) was used to examine the morphology and elemental composition of biosynthesized AgNPs in the reaction mixture.

Fourier Transform Infrared Spectroscopy (FTIR) analysis was performed on a VECTOR 22 Bruker (Germany) to identify the potential biomolecules which are responsible for reducing and capping of AgNPs.

The XRD patterns of biosynthesized AgNPs were analyzed using an X-ray diffractometer, Philips X’Pert-MPD, with tube: Co, λ: 1.78897 Å, step size: 0.02°/s, voltage: 40 kV, current: 40 mA over the 2θ range of 10–90 °C.

For FTIR and XRD analysis, freeze-dried powder of the synthesized AgNPs by freeze dryer (Christ Alpha 1- 2Dplus, Germany) was used.

### Antibacterial assay, minimum inhibitory concentration (MIC) and minimum bactericidal concentration (MBC) of AgNPs

The antibacterial activity of biosynthesized AgNPs against *P. tolaasii* Pt18 was carried out according to the Kirby–Bauer disk diffusion susceptibility test method^62^. Freshly prepared *P. tolaasii* Pt18 The bacterial suspension was adjusted to about 10^8^ CFU/ml and cultured on nutrient agar medium (Merck, Germany) using a sterile cotton swab. The 5-mm filter paper disk impregnated with AgNPs was placed on a medium. In addition, filter paper disks impregnated with silver nitrate solution (1 mM) and bacterial suspension were used as controls. The plates were then incubated at 26–27 °C and the size of the inhibition zone of bacterial growth was measured.

To determine the MIC, the freshly prepared *P. tolaasii* pt18 suspension in LB broth was adjusted to the concentration of approximately 10^6^–10^8^ CFU/ml. The microtubes were filled with 100 µl LB medium containing 10 µl of bacterial suspension and AgNPs with the final concentrations of 1000 to 1.97 µg/ml. The microtubes were incubated at 26–27 °C for 24 h and the growth of bacteria was determined by measuring the optical density (OD) at 600 nm using a UV–Vis spectrophotometer^62^. The LB media with/without bacteria alone served as positive/negative controls, respectively.

After the determination of the MIC value of the AgNPs, aliquots of 50 μl from all the microtubes which showed no visible bacterial growth were loaded on NA medium plates and incubated at 26–27 °C for 24 h. When no growth of the bacterial population was observed at the lowest concentration of AgNPs, it is termed the MBC endpoint^63^. All experiments were repeated three times.

### The effect of AgNPs on bacterial growth

The effect of biosynthesized AgNPs on the growth of *P. tolaasii* Pt18 was measured as previously described^64^. *P. tolaasii* Pt18 was grown in NB medium supplemented with biosynthesized AgNPs (7.8, 3.9, and 1.97 µg/ml) and incubated at 26–27 °C for 30 h. The growth was monitored after 6, 12, 18, 24, and 30 h by using UV–Vis spectroscopy (600 nm). Media inoculated with bacterial strain alone or AgNO_3_ (7.8 µg/ml) served as controls. The experiment was repeated three times.

### Tolaasin detoxification assay

The detoxification assay was conducted according to the procedure previously described^32^. *P. tolaasii* Pt18 strain was grown at 25 °C for 48 h in PSB (potato semi-synthetic broth) medium. The culture was centrifuged (13,000 rpm, 4 °C) and the cell-free supernatant containing tolaasin was sterilized by placing it in boiling water for 10 min. The supernatant obtained was added to a freshly prepared PSB medium (PSB-Tol). Then, biosynthesized AgNPs were added to 100 μl of PSB-Tol to a final concentration of 7.8 mg/ml. The cultures were further incubated at 26–27 °C with shaking at 110 rpm for 48 h. After centrifugation (13,000 rpm, 4 °C), supernatants were filter sterilized and 50 μl was applied onto sterile potato tuber slices. Color change of potato tuber slices from brown to blackening was recorded as the level of tolaasin detoxification. Potato slices treated with PSB + AgNO_3_, PSB, and PSB-Tol were served as controls. The experiment was repeated three times.

### Motility assay

The effect of biosynthesized AgNPs on the motility behavior of *P. tolaasii* Pt18 cells was assessed using the Agar diffusion method^65^. Freshly prepared *P. tolaasii* Pt18 culture was adjusted to a concentration of about 106 CFU/ml, and then 2 µl of the cultures were spotted onto plates containing 0.7%, 1.6%, and 0.2% of agar in King B medium mixed with AgNPs (final concentration of 7.8 µg/ml) for swarming, twitching and swimming motility, respectively. The plates were incubated at 25–26 °C. Swarming and swimming motility halo were examined after 72 h, and twitching motility was observed after 48 h. The experiments were repeated three times with three replicates. The bacteria cultured in King B medium and supplemented with AgNO_3_ were used as controls.

### Chemotaxis assay

The chemotaxis assay was performed according to the method previously described^66^. The chemotaxis buffer medium (10 mM phosphate buffer, 0.1 mM EDTA, 1 M methionine, 10 mM lactic acid, 0.35% agar, and pH 7.3) was mixed with biosynthesized AgNPs to a final concentration of 7.8 µg/ml and poured on Petri plates. The holes (10 mm in diameter) in the chemotaxis agar medium were prepared and filled with 100 µl of the mushroom caps extract. Then, a 2 µl overnight growth culture of *P. tolaasii* Pt18 was spot inoculated at a distance of 15 mm from the hole. The plates were sealed with parafilm and incubated at 25–26 °C for 48 h. The movement of *P. tolaasii* Pt18 cells toward the extract was counted as CFU/ml. Bacterial cells inoculated into chemotaxis medium and medium containing AgNO_3_ (7.8 µg/ml) were used as controls. The experiment was repeated three times.

### Biofilm formation assay

The biofilm formation ability of *P. tolaasii* Pt18 cells treated with biosynthesized AgNPs was investigated in polypropylene tubes. Ten microliters of *P. tolaasii* Pt18 (concentration of about 1 × 10^11^ CFU/ml) was added to 190 μl of LB medium supplemented with biosynthesized AgNPs (final concentration of 7.8 mg/ml) in 0.2 ml Eppendorf tubes and incubated at 26–28 °C for 24 h without shaking. The biofilm formation was quantified by the crystal violet staining method previously described^67^. In brief, 25 μl of 1% crystal violet solution was added to each tube and incubated at room temperature for 15 min. The tubes were then rinsed thoroughly with sterile water. After that, the attached cells were solubilized by the addition of 2 × 200 μl of 95% ethanol, the volume was brought to 1 ml with sterile water and the absorbance was measured at 540 nm with a spectrophotometer (SPECORD 210, Analytik Jena, Germany). Non-treated bacterial cells with AgNPs or treated with AgNO_3_ served as controls. The experiment was conducted with three replications. In addition, the stained bacterial cells were fixed and examined using an optical microscope.

### Effect of AgNPs on cell morphology

External morphological changes of the *P. tolaasii* Pt18 cells treated with biosynthesized AgNPs were examined using scanning electron microscopy (SEM)^33^. *P. tolaasii* Pt18 cells were cultured in plats containing King B medium supplemented with biosynthetic AgNPs (7.8 mg/ml). Plates were sealed with parafilm and incubated at 25–26 °C for 72 h. Afterward, the cells were collected into Eppendorf tubes, washed three times with sterilized water, centrifuged (10 min, 5200 g, 4 °C) and the pellets were stored at − 20 °C overnight. Cells were dehydrated by the freeze-drying process at − 40 °C for 3 h. Finally, the samples were coated with gold, and electron micrographs were taken using the TSCAN SEM (TSCAN SEM, TSCAN, Czechoslovakia).

### Effect of biosynthesized AgNPs on brown blotch disease development

Mushroom caps (approximately 3–4 cm in diameter) in stages 2 or 3 were harvested and placed in sterile Petri dishes. Suspension of overnight growth of *P. tolaasii* Pt18 (approximately 10^6^ CFU/ml) and biosynthesized AgNPs 15 mg/ml were sprayed onto caps immediately and after 30 min, respectively. Inoculated caps were incubated at 25 °C with 85% relative humidity for 2 days. The browning level of cap color was measured with Chroma Meter (TES135A, Taiwan) using the L, a, b scale, and ΔE was defined as √ [ΔL^2^ + Δa^2^ + Δb^2^] following the procedure previously described^68^. The color measurement was the average of three samples. Mushroom caps inoculated with *P. tolaasii* Pt18 alone, sterile water, and AgNO_3_ were used as controls.

### Statistical analysis

Statistical analysis was carried out using the statistical software SAS version 9.1. The differences between the treatments were statistically analyzed using analysis of variance (ANOVA) and followed by Duncan’s multiple range test at p = 0.05. All graphs were plotted using Excel software.

## Data Availability

All gene sequence data and bacterial strains information are deposited in NCBI database and publicly available through the web link: https://www.ncbi.nlm.nih.gov/nuccore/OP748753, https://www.ncbi.nlm.nih.gov/nuccore/ OP886707. Other data that support the findings of this study are available from the corresponding author upon reasonable request.
